# Development of a standard form for assessing research grant applications from the perspective of patients

**DOI:** 10.1186/s40900-018-0112-4

**Published:** 2018-09-03

**Authors:** Maarten de Wit, Truus Teunissen, Lieke van Houtum, Margriet Weide

**Affiliations:** 1 Department of Medical Humanities, Amsterdam Public Health (APH), VU University Medical Centre, Amsterdam, Netherlands; 2 Stichting Tools2use, Amsterdam, Netherlands; 3 Dutch Association of Health Care Funds (SGF), Amersfoort, Netherlands; 4 Dutch Diabetes Research Foundation (Diabetes Fonds), Amersfoort, Netherlands; 5 Lung Foundation Netherlands (Longfonds), Amersfoort, Netherlands

**Keywords:** Patient participation, Patient involvement, Patient engagement, Patient reviewers, Research grants assessment

## Abstract

**Background:**

Health-research funding organizations are increasingly involving patient representatives in the assessment of grant applications. However, there is no consensus on an appropriate scope or definition of the patient perspective and the eligibility of potential patient reviewers to take on this role. The aim of our study is to develop a consensus-based template for patient reviewers to assess research grant applications from the patients’ perspective. We also defined a glossary of terms and definitions to help the patient reviewers in their assessment role.

**Methods:**

Together with members of the Dutch Association of Health Care Funds (SGF) we developed an assessment form for patient reviewers following constant comparative analysis of existing review forms, a survey among all stakeholders, testing in three pilot training sessions, and a structured consensus process.

**Results:**

A small SGF working group collected and analysed 20 patient assessment forms, used by 12 health foundations and one patient organization. One systematic literature review was included. By comparing and discussing items and assessment categories in subsequent workshops, a first template form was developed. This version was electronically distributed among the members of 10 patient panels of whom 67 patient reviewers filled in the survey. A second version was then presented at a final working group meeting where consensus was reached about a template with 12 categories covering 41 items important for patients. A brochure for patient reviewers, a guide for panel coordinators and a glossary were developed to accompany future implementation of the template.

**Conclusions:**

A template for patient reviewers to assess research grant applications is now available, based on the consensus of 21 Dutch health foundations.

**Electronic supplementary material:**

The online version of this article (10.1186/s40900-018-0112-4) contains supplementary material, which is available to authorized users.

## Plain English summary

Organisations that fund research value the opinion of patients. To decide which research should be funded they may invite patients to assist in assessing the relevance and feasibility of a research proposal from the perspective of patients. People who have experienced knowledge of living with a health condition and who assess research applications, are called patient reviewers. However, it is not always clear how they should assess a research proposal. This study aimed to develop a standard assessment form that guides patient reviewers to form an opinion about the relevance, acceptability and feasibility of a research proposal. Together with members of the Dutch Association of Health Care Funds (SGF) we developed this form by comparing 20 different patient assessment forms. In addition we sent out a questionnaire to gather the opinion and experiences of patient reviewers. Sixty-seven patient reviewers filled in the questionnaire. Their responses resulted in a form with 12 categories covering 41 items important to patients. The categories and items deal with, among others, the relevance of the study for patients and society, inclusion criteria (who are eligible for participation), safety, burden and risks for patients, privacy, communication and opportunities for public and patient engagement. A brochure for patient reviewers and a guide for employees of research funding organisations were developed to support the use of the standard assessment form. We hope that many funding organisations will use this form which makes it easier for patient reviewers to express their opinion about research proposals.

## Background

Many health funding organizations involve patients in the merit review of research grant applications [1]. The role of patients as reviewers aims to assess grant proposals from the perspectives of end-users, often persons with first-hand experience of the illness or limitation or a person representing the target group under consideration.

Over the years organizations have developed different forms and criteria to guide patient reviewers in the assessment of grant proposals. Guidance for patients is limited, although some international examples of assessment forms are publicly available [2]. The British Medical Journal has published a document for patient reviewers of scientific manuscripts on their website [3]. And in 2014 Teunissen explored relevant criteria directly derived from people with a chronic condition [4]. The scope of this study was not limited to the area of research but also included the areas of health quality improvement and health policy [5]. From that study we know that criteria such as relevance, burden to study participants, privacy protection, proposed outcome measures (endpoints), inclusion and exclusion criteria, patient engagement, communication with study participants and implementation and dissemination of research findings are important items for patients.

Despite the existence of some guidance for patient reviewers, major challenges still wait to be addressed. A recent publication evaluating the public involvement in the context of health technology assessment clearly found limitations of its procedures and impact [6]. First of all, there is no consensus on the scope or items that patients should include in their review of applications. Many organizations have developed their own templates and procedures, although they are scarcely published and therefore difficult to study. We know however that the scope, layout and content of these templates as well as the patient review process vary widely, depending on the health condition at stake, the type of research, and the level of professionalism of the organization or their patient panel. Some patient reviewers work individually, others work in small groups. When meetings take place, they can be face-to-face or virtual. The results can be written in a report, a consensus statement or a table with final scores. Some panel members assess a limited number of grant applications, others assess all applications. As a result of the heterogeneity of assessment forms and procedures, patient reviewers in the Netherland reported the need for standardization and more detailed guidance for assessing grant applications from a patient perspective.

A second challenge is the lack of consensus on who can be eligible for becoming a patient reviewer. Organizations differ in their recruitment and selection process. Some organizations invite only people with first-hand experience of a health condition while others seek broader stakeholder involvement, including carers, family members, representatives of patient organisations or even health professionals. Depending on the composition of the patient panel, the representativeness of the patient perspective may become arbitrary.

Research foundations may allocate different roles to stakeholders in their decision making process [7]. In some foundations all stakeholders are involved in one Medical Advisory Board with decisive power, while other organisations have separated the assessment of quality from the assessment of relevance, often called societal impact. The SGF is a national association comprising 21 independent health care funds in the Netherlands (Table 1). They represent the interests of around 5 million individual donators and 800.000 volunteers. Fund raising for scientific health research is one of their major responsibilities.

**Table 1:** Involvement of SGF member organizations and external stakeholders in the development of the standard form for assessing research grant applications from the patients’ perspective

SGF Member Organisations	Patient Review Form	Survey	Two SGF Workshops	Three Pilot Trainings	SGF Committee
Johanna Kinderfonds (Children)					
Epilepsy Foundation					
Rehabilitation Foundation					
Lung Foundation Netherlands	●	●	●	●	●
MIND/ Fonds Psychische Gezondheid		●		●	●
Dutch Diabetes Research Foundation	●	●	●		●
Dutch Alzheimer’s Society	●	●			●
Dutch Digestive Foundation	●	●		●	●
Dutch Cystic Fibrosis Foundation	●	●	●		●
Prinses Beatrix Foundation		●		●	●
Dutch Heart Foundation	●	●	●		●
Brain Foundation Netherlands					
MS Research Foundation					
Dutch Burns Foundation			●	●	●
Dutch Kidney Foundation	●		●		●
Dutch Cancer Society	●	●		●	●
Dutch Arthritis Foundation	●		●		●
Aids Foundation					
Fonds Verstandelijke gehandicapten					
Dutch ALS Foundation					
Dutch Thrombosis Foundation					
External Stakeholder Organisations					
Dutch Patient Federation^a^	●	●	●	●	●
PGOsupport^a^			●	●	●
ZonMW^a^	●		●		●
BVN/BOOG (Breast Cancer)	●			●	

^a^ Participation in the committee as invited advisor; ALS (Amyotrofe Laterale Sclerose); BOOG (Breast Cancer Research Group); BVN (Dutch Breast Cancer Patient Association); MS (Multiple Sclerosis); SGF (Dutch Association of Health Care Funds); ZonMW (Dutch Organisation for Health Research and Innovation)

The objective of this study is to present the methods and outcomes of the process of developing a form for the assessment of research grant applications from the perspective of patients and to outline the next steps for validating and implementing this standard form among patient reviewers and health research foundations.

## Methods

### Working group

The initiative for elaborating a template form for patient reviewers was taken by the committee for Patient Participation of the Dutch Association of Health Care Funds (SGF). This committee contains 11 members. They established a small working group with proportional representation of patient experts (MdW and TT, both holding a PhD in collaborative research), and research coordinators, responsible for organizing the patient perspective in the grant selection process of their organisations (LvH, MW). During 15 months the working group followed a process of co-production. The project proposal, interim results and final documents were presented and discussed at every meeting of the SGF committee.

### Stakeholder involvement

During the second half of the study three stakeholder organisations were consulted regularly because of their important role in implementing recommendations for patient reviewers. PGOsupport, responsible for education of patient representatives in the Netherlands, ZonMW, the Dutch Organization for Health Research and Innovation and Patient Federation Netherlands, commissioned with the coordination of the patient panel for reviewing ZonMW grant applications. All three stakeholder organisations participated in two workshops and one SGF committee meeting. In addition to this, PGOsupport provided an opportunity to test draft versions of the assessment form in three face-to-face pilot trainings for patient reviewers. The Patient Federation participated in the recruitment of respondents for the survey. Table 1 shows the level of involvement of all SGF member organisations and the three external stakeholders.

### Development of the form

**Fig. 1:**
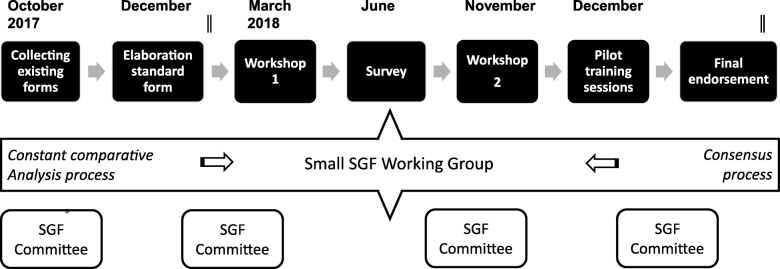
The development process of the standard form for assessing research grant applications form patients’ perspectives

The small working group followed an iterative consensus based development process including multiple forms of data collection and validation (Fig. 1). Based on the criteria core set published by Teunissen e.a [8]. and patient assessment forms of SGF members, we followed a constant comparative method to compose an initial list of items relevant for capturing patients’ perspectives on scientific research proposals. The constant comparative method is a key component of grounded theory and provides a systematic approach through which subjective values and interpretations can be structured [9]. By collecting data, breaking down in units, comparing, discussing and grouping items into recurring categories, a preliminary version was agreed by the working group. Disagreements were resolved through discussion until a consensus was reached. Development of the standard form required subsequent rounds of data collection and analysis until the final version was agreed. We complemented the development by an update of the systematic literature review of relevant patient criteria in health research, health care innovation and health care policies [10]. Findings and decisions were reported during each step in the development process.

The preliminary version of the item list was discussed in a wider group of stakeholders during a first workshop on patient engagement organized by SGF in collaboration with three external stakeholder organisations (Spring 2017) and in a second World Café workshop organized for all SGF members and the three stakeholder organizations (Autumn 2017).

Patient reviewers of SGF member organizations were consulted through an electronic survey (SurveyMonkey) to give feedback on the relevance, completeness and clarity of assessment categories and sub-questions. To explore validated scoring methods two members of the core group consulted the Dutch Julius Centre, an expertise centre for outcome research, to obtain advice on the preferred method of scoring. Each SGF committee member organization and the Patient Federation were asked to send the survey randomly to 20 patient reviewers. Ethical approval for the survey was not sought. No personal or medical information was collected other than membership of a (SGF) patient reviewer panel (22 options) and role in the review process (6 options: patient, family member, carer, patient representative, SGF staff person, other). Validation of the form took place during three training workshops, organized by PGOsupport for members of patient reviewers panels (Table 2). Consensus was achieved through intense deliberation among the SGF committee members. After full consensus on the final draft by the SGF committee, the SGF board endorsed the form in December 2017 including the two accompanying guides for patient reviewers and for organizations.

**Table 2:** Training workshop participants

	Training workshop 1	Training workshop 2	Training workshop 3	TOTAL
	April 20, 2017	May 19, 2017	December 8,2017	
TOTAL NUMBER	12	7	12	31
Female	8	2	8	18
New^a^	5^a^	7	5^a^	17
BACKGROUND				
Patient representative	7	3	9	19
Family or informal carer	2	1	2	5
Staff member	1		1	2
Public representative	2	3		5
CONDITION				
Parkinson	3			3
Mental Health	3		3	6
Lung	2			2
Cancer			5	5
Muscles			1	1
Obesitas			1	1
Burn injuries			2	2
Patient Federation	4	7		11

^a^Estimate

## Results

### Development of the standard form

The small working group received 12 assessment forms, six application forms and two templates for lay summaries. Following an approach of constant comparative analysis we developed a first list of 51 items capturing 10 categories.

During a first one-hour workshop (March 2017) participants emphasized the importance of streamlining assessment criteria among research funding organizations and discussed the requirements of a framework of mandatory and optional items to assist patient reviewers in their work as reviewers. It became clear that the context is important for selecting the relevant items. The type of research may vary and influence the selection of relevant items. For example, to assess a project idea patient reviewers should focus on the relevance of the research question, the potential added value for patients and whether the proposed research methods are feasible from the perspective of patients. In contrast to this, assessing a full research protocol requires more attention for details about burden and risks for patients, proposed outcome measures, inclusion and exclusion criteria and the way patients will be informed and involved before, during and after the study. The group also felt that assessing basic research protocols requires other categories and items than translational, clinical or social health research. For this reason we distinguish recommended and optional items for five different types of research applications (Additional file 1: Table S3). At a later stage patient reviewers expressed their wish to be involved in the selection of recommended and optional items, depending on the disease, their organization or type of research call.

Finally, the participants indicated the need to provide guidance to researchers and research organizations how to implement the form in daily practice and asked for flexibility to adapt the list to the characteristics of their organisation. This should for instance be the case for weighing the relevance of the assessment criteria. They agreed that this is not the same for all the funding organizations.

In addition to the original systematic literature review (SLR) conducted by Teunissen e.a [8], .we obtained access to the data of a SLR update conducted by the Free University of Amsterdam [10]. The preliminary version of the item list was mapped against the new SLR findings that confirmed the adequacy of the generated categories and the respective items.

A recurrent point of discussion was the relevance of reviewing outcome domains and measurement tools (endpoints) by patients. During different consultation rounds among all stakeholder groups, including patients and panel coordinators, different opinions arose as to whether outcomes are an item on which patient reviewers can provide meaningful input. After subsequent deliberations and proposal, it was decided to keep ‘outcomes’ and ‘instruments’ as an important item and to provide clear explanations and examples in the guide for patient reviewers.

The survey was filled in by 67 patient reviewers. Their characteristics are presented in Tables 3 and 4. As a result of the survey findings we created, among other adjustments, a separate category called ‘representativeness’ with four items. Respondents also proposed more items related to safety and ethical considerations. For this reason we changed the heading of the category ‘Patient Information Sheet’ into ‘Ethics and Safety’ and added one item.

**Table 3:** Membership survey participants (*n* = 67)

Membership organisation	*N* (%)
Dutch Alzheimer’s Society	5 (8)
Dutch Diabetes Research Foundation	1 (2)
MIND/ Fonds Psychische Gezondheid	11 (15)
Dutch Heart Foundation	3 (5)
Lung Foundation Netherlands	13 (19)
Dutch Digestive Foundation	3 (5)
Dutch Cystic Fibrosis Foundation	7 (10)
Dutch Arthritis Foundation	4 (6)
Prinses Beatrix Foundation	1 (2)
Dutch Patient Federation	19 (29)
Total	67

**Table 4:** Role survey participants (*n* = 67)

Role	*N* (%)
Patient	39 (58)
Proxi / Family member	9 (14)
Carer	2 (3)
Patient representative (advocate)	13 (19)
SGF staff member	0 (0)
Other	4 (6)

During a third training session where we piloted the preliminary assessment form, new patient reviewers asked how they should review research proposals that they had seen before. This caused an intense discussion about confidentiality and dealing with a conflict of interest. For some patient reviewers it was an eye-opener that they should not get involved in assessing grant application for which they had provided input or advice, or for which they were even co-applicants or collaborative partners. Our form clearly missed an introductory item checking for potential conflicts of interest. We corrected this omission in the final draft version.

This final standard form (Additional file 1: Table S3), includes 12 categories and 41 items, was discussed at the last SGF committee meeting and unanimously approved. One Working Group representative presented the draft list during the SGF General Assembly where it was also unanimously endorsed. Implementation has started in January 2018 by 3 SGF member organisations. Monitoring of the application of the standard form is on the agenda of every SGF Patient Participation committee meeting and formal evaluation of the form is planned after 1 year.

### Guide for patient reviewers

Based on the feedback of patient reviewers from the survey and the three pilot training sessions, we developed a guide for patient reviewers (Additional file 2). The guide explains the four assessment scores and the requirement of confidentiality when assessing research applications. It also clarifies the main categories and gives definitions of some of the terms such as quality of life, social participation, societal impact, cost effectiveness, inclusion and exclusion criteria, study participant, consultation, patient research partner, diversity and informed consent. For the concept of “outcomes” the guide provides a comprehensive explanation and additional examples. The guide is an integral part of the training for patient reviewers.

### Guide for organisations

From the feedback from professionals working for health foundations it became apparent that panel coordinators requires clarification on the use of the form in daily practice. We therefore composed a guide for people responsible for organizing the patient perspective in grant assessment procedures (Additional file 3). This guide contains recommendations for implementing the form and how to select items that are tailored to the characteristics of the organization or the research program, preferably with active involvement of the patient reviewers. There is a paragraph on the nomenclature around the concept of patient reviewer. Organizations have to determine who is eligible to take on the role of a patient reviewer. Practice shows that there is a variety of profiles for patient reviewers, diverging from people with first-hand experience of living with a condition or illness to family members, carers, patient advocates or even health professionals. The guide contains descriptions of these different profiles and the implications for the selection process. Depending on the competences of the panel members, the assessment process should require comprehensive summaries in lay language. In countries for which English is not the first language, the organization should decide whether they ask researchers to translate lay summaries when written in English. Panel coordinators are also suggested to provide feedback to patient reviewers after the assessment process, not only on the impact of their input on the research applications but also on the final decisions made by the funding organisation.

The guide for organisations recommends providing face-2-face training to patient reviewers, introducing the form and guides, and introducing points to consider when giving feedback to researchers. In addition we recommend providing professional support of patient reviewers by taking care of logistics [11], for instance by providing the grant applications in time; For those who prefer a printed version, sending these by post; Sufficient time for reviewing; Fast reimbursement of expenses and sharing literature on request. This will enhance motivation and continuity of the panel members and make their work less burdensome. Finally, the guide recommends providing support and guidance to researchers as well.

## Discussion

We developed a standard assessment form for reviewing research grant applications from the patients’ perspective. The form is unanimously endorsed by the largest health care funds in the Netherlands and comprises 12 categories capturing 41 items. Research foundations and patient organizations are expected to adapt the form to the characteristics of their own organization or research program. A guide for patient reviewers as well as a guide for research coordinators accompanies the form.

Implementation of the form has started. Preliminary findings of the Dutch patient panel of the Cystic Fibrosis foundation are positive. Also ZonMW started the implementation of the form in a large program on translational research. We expect that the form can be used in other countries and contexts where patients are involved in the prioritization and review of research grant applications. Comparison of the SGF form with those from NIHR in the UK [12] makes clear that different views on patient involvement in research grant application assessment exist. While in the Netherlands the role of patients and patient organisations is prominent, in the UK a more broad approach of public and patient involvement is sought. The same is true for the topics that are included in assessment forms. Topics that patient reviewers in the Netherlands found important and are included in NIHR documents are ‘relevance of the proposed research’, ‘plain summary’, ‘expected impact’ and ‘involvement of patients’. However, NIHR topics such as ‘research design’, ‘work plan and management’ and ‘strength of the research team’ were not perceived as typical patient relevant assessment criteria by the Dutch patient reviewers. Within the SGF these topics seems more relevant to be assessed by peer reviewers.

A first challenge of implementation is the tendency that we have observed over the years to recruit and select patient reviewers who are higher-educated, middle-aged, more than often female and usually Caucasian [13]. They also may not represent the patients with more severe disease activity. This stimulates the debate about representation: how well does the feedback of patient reviewers reflect the perspective of all patients? In the Netherlands several funding agencies enable patient reviewers to assess grant applications in small groups of three or four. Working as a team has multiple advantages. It stimulates intense interaction between reviewers about the relevance, values and design of research proposals [14]. These discussions often lead to more in-depth and diverse feedback because people may represent different stages or types of disease or different experiences with health care.

Over the years the perception of the role of patients as reviewers has evolved. They are increasingly perceived as patient experts who provide advice to researchers how they should improve their proposal to better meet preferences and expectations of patients, including recommendations for patient involvement in the study to ensure that these preferences and expectations are not lost along the way. This implies that their primary role is not to be fully ‘representative’ for the target audience or that they should act on behalf of a patient organization. Ideally patient reviewers approach applications with an open mind and fulfil their role without being bound by a mandate or agenda of any organization with which they may be affiliated.

Another important challenge of implementation is the commitment of researchers to proactively provide information that patients find important in a language that patient reviewers can understand. They should have the ability to anticipate the feedback of patients. For most researchers this ability does not come automatically. For this reason researchers, in addition to patient reviewers, need to be informed and prepared as well. This can be done through clear guidance through the research application form or through coaching or training [15]. The organization could also consider offering support for researchers who want to receive advice on how to capture the patient perspective in their research proposal.

The lack of involvement of researchers in the development of the form can be seen as a limitation of the study. However, we assume that the representatives of the SGF member organisations have taken the interest of researchers they fund into account to prevent the inclusion of items that are not feasible to address in a research protocol. On the other hand, we also believe that the perspective of researchers is sufficiently captured in the existing review forms that are used by funding organizations for professional peer review.

The concept of co-production ensured that all patient reviewers’ input was maintained throughout the development process. The persistent feedback from the members of the SGF committee members created a strong feeling of ownership over the standard form. Next steps in the implementation of the form are the development of a template for public summaries of research proposals as well as a template for research grant applications. Researchers and patient reviewers will benefit hugely when funding agencies streamline their grant application procedures by using standard forms that are consistent, using the same terminology and having the same order of categories and items. Patient reviewers often complain that feedback forms and application forms are not consistent, which makes the review process challenging. Researchers will benefit from more consistent forms when they don’t have to rewrite their proposal when applying to another funding organization. Finally, it is desirable that also the scoring methods of professional reviewers and patient reviewers are streamlined, enabling decision bodies to compare the assessment of both stakeholders, using similar scorings.

## Conclusion

Over the years research foundations have developed a variety of forms for researchers to apply for funding. Since patient representatives are involved as reviewers, many assessment forms are developed that are often not consistent with the existing application forms. For this reason the Dutch associations of 21 independent health research foundations took the initiative to develop a standard form for assessing research proposal from the perspective of patients. This standard form supports patients in their role as reviewers and enhances the quality of their assessment as well as their feedback to researchers. The standard form is also helpful for researchers who are writing a research grant application. It informs them about the criteria that are important to patients. Finally the form stimulates research institutes and funders to consider patient and public involvement more seriously. Because the standard form is endorsed by many health research organisations in the Netherlands, education of lay persons can be uniformed and, together with broad implementation of the form, lead to more meaningful and effective research assessment procedures.

## Additional file

Additional file 1: **Table S3**. Standard form for the assessment of research grant applications from the patients’ perspective *. (PDF 251 kb)

Additional file 2: English guide for patient reviewers. (PDF 321 kb)

Additional file 3: English guide for organisations. (PDF 310 kb)

Additional file 4: English standard form for patient reviewers. (PDF 227 kb)

Additional file 5: English standard form for organisations. (PDF 248 kb)

## Abbreviations

NICE: National Institute for Health and Care Excellence
NIHR: National Institute for Health Research
PGOsupport: Dutch Independent Network Organisation for Patient and Client Organisations
SGF: Dutch Association of 21 independent Health Care Funds
SLR: Systematic Literature Review
ZonMW: Dutch Organization for Health Research and Innovation
