# Supplementary material for: Development of a standard form for assessing research grant applications from the perspective of patients
Source: Res Involv Engagem. 2018 Sep 3;4:27. doi: 10.1186/s40900-018-0112-4 (PMC6120065; doi:10.1186/s40900-018-0112-4)
Supplement: Supplementary file 2 — English guide for patient reviewers. (PDF 321 kb) [file 40900_2018_112_MOESM2_ESM.pdf]

# Guide for patient reviewers

**Introduction to the SGF form for the assessment of research grant applications from the patients' perspective**

This brochure describes the content of the SGF form for the assessment of research proposals by patient reviewers.

It is meant for people who assess a research proposal from the perspective of the target group, also called the patients' or users' perspective.

The assessment form comprises 12 categories. Below we explain the sub-questions and the used terminology.
For the first eleven questions 4 options to answer exist: good, sufficient, moderate and insufficient.

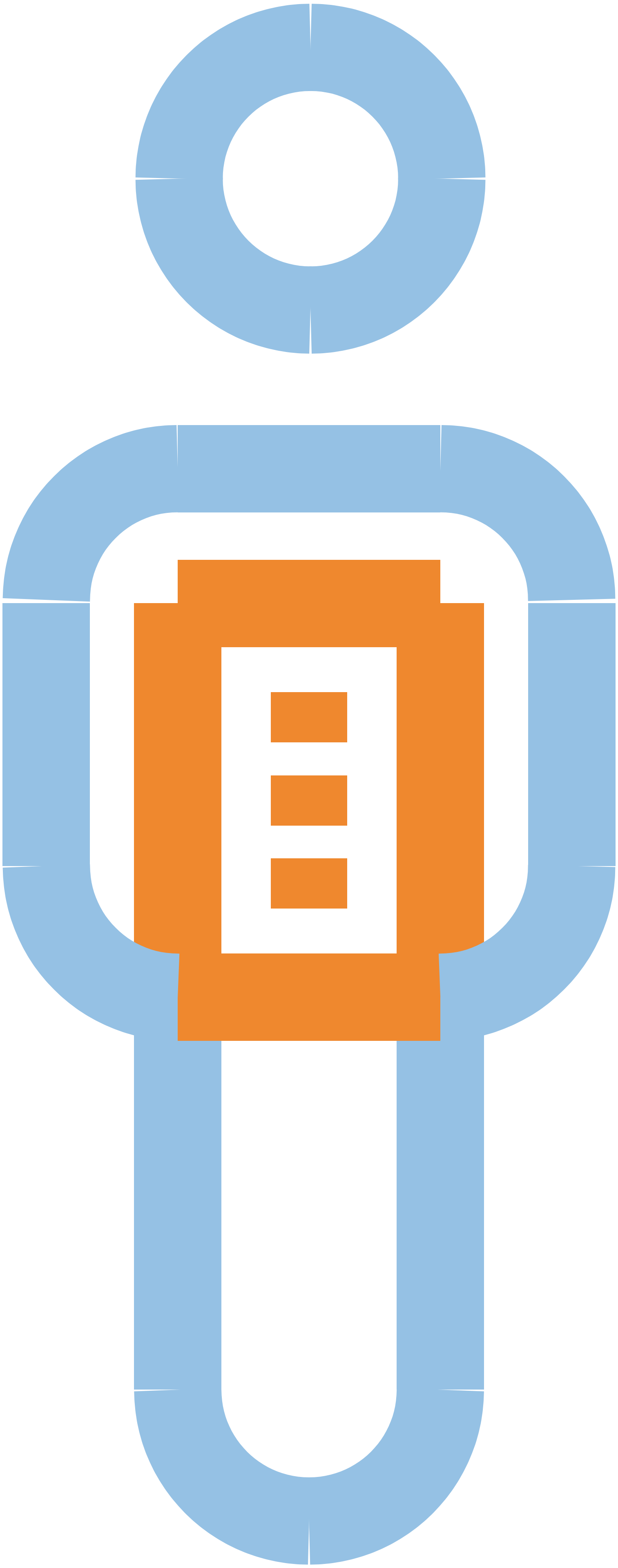

Samenwerkende GezondheidsFondsen
SGF

| | |
|---|---|
| **Good (G)** | **You are satisfied with information in the application;** |
| **Sufficient (S)** | **Some information is lacking or you wish some minor adjustments or clarifications;** |
| **Moderate (M)** | **Much information is lacking or you wish some major adjustments or clarifications;** |
| **Insufficient (I)** | **You are not satisfied with the information in the application because important information is lacking or because you think the information is not correct.** |

Try to explain your answer concisely. Use the opportunity of n.a. (not applicable) only if the question cannot be answered, for instance because there is no lay summary (question 1) or Patient Information Form (question 9). Also for category 3 (relevance for society) it is not always possible to answer all sub-questions and you might want to choose for n.a..

# Involvement and confidentiality

If you answered ‘yes’ to the question about your involvement, you should consider whether you feel sufficiently able to objectively assess this application. For reasons of transparency and to avoid conflicts of interest, it might be better to leave the assessment to other patient reviewers who are not involved in the application.

Furthermore, it is important that you are aware of the fact that this research application is provided to you in confidence. For the applicant of this research proposal it is important to be ensured that the content of the application is not shared with others who may gain personal benefit from this. It is expected from you that you don’t speak about this application in the presence of third parties nor that you circulate the text among others. This means that your assessment is anonymous and that others will handle carefully with your assessment.

# 1. Lay summary

This item is about the question whether you think the summary is clearly written. That means, is the summary easy to read, comprehensible and sufficiently complete to come to a first judgement of the relevance of the application. Easy to read means for instance that sentences are short and jargon is avoided. This summary is primarily written for patient reviewers.

# 2. Relevance for the target group

To what extend is the topic relevant for people that belong to the target group of this research? If a research agenda from the perspective of patients exists, does this application meets one of the priorities of the target group?

## 2.1 Informal carers

Important persons in the direct environment of the patient, often a partner or other relative.

## 2.2 Self-efficacy

The ability of people to live their life as much as possible independent from others. Within the context of mental health this is also described as “working (together) for recovery”.

## 2.3 Quality of Life

This form uses the official definition and explanation of the World Health Organization (WHO). Quality of life means here ‘*the perception of individuals of their position in life in the context of the culture and value systems in which they live and in relation to their goals, expectations, standards and concerns.*
Quality of life has objective and subjective aspects. Objective aspects can be observed by others and eventually measured such as decreased life duration, kidney failures, a skin condition or a physical limitation. Subjective aspects comprise judgements that a person makes about his or her own well-being. They are related to the attempt to achieve personal goals (hobby’s, preserving personal relationships etc.). Several sub-questions in this form are part of the concept of Quality of Life.
It is important to emphasize that it is not only about physical, but also about psychological and social aspects of daily life.

## 2.6 Quality of care

Think not only about the expansion of medical possibilities, but also about accessibility of care, services, attitudes and improvement of efficiency in care that can, for instance, lead to shorter waiting lists or better multidisciplinary care.

## 2.8 and 2.9 Outcome measures

An outcome or endpoint is the result of a measurement of the effect of an intervention (most often in a clinical study. Often used outcomes are for example survival (mortality), pain, fatigue, physical function, self-efficacy (social) participation, a laboratory test (sedimentation rate in blood), the result of a MRI or a Quality of Life questionnaire.
It is important that research is focussed on outcomes that matter to patients and that instruments are used that really measure these outcomes. Box 1 contains an example of the importance of adequate instruments to measure an outcome and why the opinion of patient reviewers matter.

### BOX 1

#### OUTCOME MEASURES IN DUCHENNE DISEASE RESEARCH

Duchenne is a rare disease that can occur in boys at a young age. Physical endurance is the usual outcome measure in clinical research and is measured with a 6-minute walking test. New and better treatments extend the lifespan of children with this disease, but in that period they can no longer walk and are then dependent on a wheelchair. Patient reviewers, in this case also parents of children with Duchenne, have therefore raised the question of how relevant the walking test is for teenagers who do all their daily activities from a wheelchair. For them, the maintenance of good hand function in order to remain mobile and independent is important. At a later stage, eating independently or breathing without help become important outcome measures of medical treatment. Patient reviewers indicated that the relevance of an outcome measure strongly depends on the phase of the disease. They therefore no longer consider a uniform outcome measure acceptable.

# 3. Relevance for society

To what extent does this research contribute to, for example, better prevention or faster diagnostics, lower costs for health care, more people who can participate in the labor market or less burden on informal caregivers? In these cases there is an indirect interest for the individual or the (potential) target group and the emphasis is on the importance for society. These are general questions about health care. These aspects have a major impact on society and are therefore also relevant for healthy people. An example is research into the desirability of a national screening program for certain health risks. Or research into the cost savings in health care through more prevention.

## 3.1 Social participation

The extent to which people participate in society. This can be in the form of voluntary work in the neighborhood or at school, but also by being a guest parent, a member of an association or performing paid work. Preventing social exclusion, social isolation, reducing social differences.

## 3.4 Understanding of the life with an illness or health condition

Many people suffer from the misunderstanding in society for certain diseases or limitations. Research can contribute to changing the image of a particular target group. By providing information and education, people can change their view on that condition (for example, as dependent and non-existent), so that there will be less stigmatization and exclusion.

## 3.5 Cost effectiveness

A lot of clinical research is accompanied by questions about the cost-effectiveness of an existing or new intervention. There is a general awareness that managing healthcare costs is a responsibility of all parties involved in society, including patient interest groups. Good research is necessary to arrive at fair assessments of how the benefits of an intervention are in proportion to the costs of that intervention. Of course, patient reviewers should look beyond the costs of health care only: If a costly treatment results in someone being able to start working and falling back less quickly, the costs of the healthcare may be higher, but the overall costs to society are lower.

# 4. Risks for study participants

What are the potential risks for the participants? This concerns, for example, risks of known but also unknown side effects of a (new) treatment. Is the difference between the new treatment and the existing treatment ('usual care') well described? Risk may also relate to the chance of dropping out of work or school due to adverse events, or to the consequences of discontinuing a standard treatment. Does the researcher mention measures to monitor, reduce or prevent side effects?

Always include the risks of an existing treatment in your assessment. After all, the question is whether the extra risks of participating in the research are acceptable in relation to the risks of the existing treatment.

**Study participant.** *A person who takes part as a participant in a research study.*

# 5. Burden for study participants

What is the burden for study participants? For the question "Do you find the burden for participants is acceptable?" you can think of, for example, the intensity of the treatment (surgery, radiation, medication, biopsies), number of questionnaires, duration of interviews, physical or psychological examinations, frequency and duration of hospital visits, disqualifications, diets, side effects or the total duration of the study. Whether or not the (extra) burden is acceptable also depends on your opinion on the relevance of the research and the expected outcomes. They must be in proportion with each other.

# 6. Feasibility of the research

This includes, among other things, whether the research goals or the working plan are realistic.

## 6.2 Collaboration

Sometimes it is important that there are good collaborative partnerships in a research project, for example when it comes to rare diseases. Then it is obvious to work together with multiple research centers (multi-center study) or even to establish international research consortia. Only in this way a sufficient number of participants can be recruited. Collaboration can also relate to an appropriate composition of a research team: have the good expertise's been brought together? Finally, collaboration can also mean that important parties (stakeholders) are involved in research, for example healthcare professionals, but also advocacy groups of the target population of this research proposal, for example patient organizations.

## 6.3 Sufficient participants

Is there a realistic estimate of the recruitment of sufficient study participants? This question is not easy to answer. For example, consider possible objections from patents against participation due to preference for certain treatment or the chance to end up in a control group instead of the intervention group. Sometimes it can be useful to ask the question: "Would you personally participate in the research as a participant if you were part of the target group of this research? Why yes/no?"

# 7. Patient involvement

To what extent are patients and / or their representatives involved in the entire research process? Think for example of the involvement of patients in the role of research partners. Is there also attention for training, guidance, support and appreciation for patient representatives? Regarding the budget: Consider also eg a meeting allowance, travel allowance and training of patient research partners.

There are examples where patient research partners are financially compensated for their time. If there is no public or patient involvement in the research, is this sufficiently explained by the applicants?

**Patient representative.** A person who is committed to the collective advocacy for a certain target group. Figure 1 (see below) indicates which people can take on this role: patient experts, family or close relatives, informal caregivers or representatives of a special interest organization.

**Patient research partner.** People who actively contribute to the development and execution of scientific research from the perspective of the target group.

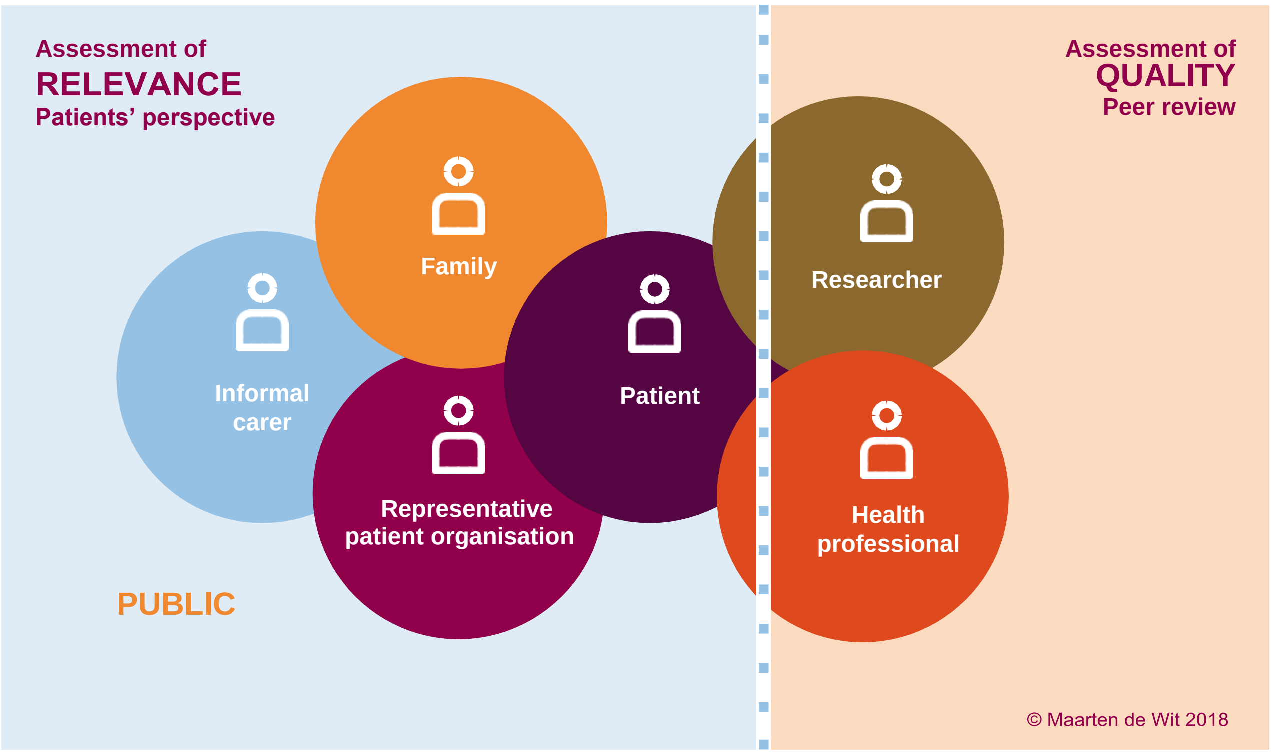


Figure 1

# 8. Representativity

Patient reviewers can play an important role in ensuring representativeness of the research. This can be done by looking critically at the questions that concern diversity, the target group of the research (inclusion and exclusion criteria) and the patient representatives involved.

## 8.1 Diversity

Attention to demographic differences (such as between men and women or difference in age), geographical differences (such as between cities and rural areas) and ethnic and socio-economic differences (such as educational level or origin). Sometimes the way of recruiting also brings along an undesirable form of selection. A university hospital, after all, attracts a different patient population than a GP practice. The question is whether there is no unwanted selection (bias) by the method of recruitment, as a result of which important groups are excluded.

## 8.2 Inclusion and exclusion criteria

Criteria to enter a research study as a participant such as diagnosis, disease activity, stage or duration of disease, control of the English language, comorbidities or experience with other treatments. Here too it is questionable whether important groups of participants are not excluded by the set criteria. After all, we want the study participants to be a good reflection of the target population.

### 8.3 Representativity patient representatives

In scientific research representativity is an important criterion for the validity (reliability) of certain findings. Full representativeness is not feasible in most cases, but there are ways to strive for this. The patients' perspective can be integrated into research in two ways, preferably in combination.

1. **Consultation**

   Individual participation of patients or their representatives without having influence on the goal and design of the research. The involvement takes place in the context of collecting data. That is why there is one-way communication: from the patient to the researcher. This form of participation can take place in the role of, for example, a participant in a study (often referred to as 'subject') or respondent.

2. **Collaboration**

   Collective representation of interests by patient experts or patient representatives who are involved in formulating the goal and design of the study. There is a partnership in which communication runs in two directions. This form of participation can take place in the role of, for example, research partner, patient reviewer or advisor.

Question 8.3 relates to the representativeness of the patient (representatives). Of course, these people can never represent the entire target population, but it is important that they adequately reflect the target group that this research focuses on. The task of the patient (representatives) is, among other things, to ensure that the patient's perspective is not lost during the different phases of the research, eg by contributing information or by giving suggestions on how this perspective can be obtained.

## 9. Ethics and safety

When applying for a clinical research grant, an ethical, legal and societal assessment is necessary in addition to an opinion from the patients' perspective. For more information, consult the website: www.ccmo.nl

All clinical research (also referred to as 'human-bound research') must be approved by an accredited committee such as the Central Committee on Human Subjects (CCMO) or the Medical Ethics Assessment Committee (METC). One of the conditions for consent is a Patient Information Form (PIF) approved by the METC or CCMO. There is a uniform PIF format that all researchers should adhere to. This format prescribes, among other things, that the PIF must have a fixed structure and may not be promotional. If it concerns a draft PIF, the feedback from patient reviewers can help the researcher to improve the PIF.

In some cases this part is not relevant, for example if there are no study participants or if it concerns a project idea. If the PIF and / or informed consent form is not available, please fill in "not applicable" (n.a.).

### 9.1 Understandable PIF

The following applies to the question of whether the PIF is comprehensible: Is clear language used? Is the text written in simple Dutch so that it can be read and understood by 95% of the population?"

### 9.2 Relevance PIF

When asking whether the PIF is correct and complete, you can think of communication with the care providers of the study participants. Are the GP, specialist or home care informed about the implications of participating in the research? Or are the risks and burden for the study participants well described, for example, is there a chance of dropping out of work, study or social obligations? Are study participants with limited health skills taken into account?

### 9.3 Informed consent

Study participants must agree in writing to participation in the study. To this end they should be sufficiently informed about the conditions under which participation takes place (PIF). This is regulated by law. For example, it should always be mentioned that participants

- are not obliged to participate (decide for yourself),
- have sufficient time to consider whether they want to participate (reflection time),
- may stop research at any time without giving any reason and without consequences for the regular treatment,
- be insured if they unexpectedly experience adverse consequences,
- can consult with an independent expert (confidential counselor) for questions and complaints

Although you as a patient reviewer may assume that the METC supervises compliance with these conditions, you can ask additional questions about these legal rights and obligations of the study participant.

Is it clear that the participants are asked to consent to participation in the research? Do the participants have sufficient freedom of choice whether or not to participate and is this clearly communicated? Do they also have enough time to read informed consent or discuss it with another person?

### 9.4 Confidentiality

Is it clear how the data of participants is handled and for how long data is stored? Is sufficient consideration taken of the privacy of the study participants in relation to their vulnerability?

## 10. Communication

This is about communication with both the study participants and people outside the research. Within the study, it concerns the study participants or their representatives. For example, they can also be family members. In your opinion, are the proposed forms of communication appropriate?

Outside the research it can be communication with patient research partners, patient representatives and the patient advocacy groups, or the dissemination of the results to the target population (end users). How do the researchers ensure that everyone for whom the results are relevant (also eg care providers, professional associations or health insurers) can take note of the results? Will results be available in a public version? Is sufficient use made of new media?

## 11. Implementation of research

How can the results of this study be implemented in daily practice? For instance, think about the consequences for existing guidelines and treatment protocols, the development or adjustment of an app, a care programme or a decision aid. Does the project team has experience with implementation or did they involve an implementation expert? Sometimes applicants provide a realistic plan for implementation and describe potential obstacles such as resistance in current practices. Sometimes relevant stakeholders are involved in the study from an early stage. Is collaboration with a patient organization foreseen? If you are aware of opportunities for implementation, you might want to write them down as suggestions. In some cases follow-up research rather than implementation is necessary.

## 12. Final Judgement

With a strong explanation or arguments from the patients' perspective you can convince both the researcher and the grant committee about your opinion. Therefore describe clearly what your overall view is on the proposal. What are strong elements and where are opportunities to improve the application? If one element is pivotal in your assessment, you might want to highlight this in your explanation.
For grant committees or boards of health foundations it is desirable that patient reviewers also make a clear and transparent prioritization when more than one application is assessed. For this reason it is important that you make a sufficiently clear distinction between proposals that you would like to see carried out (approve) and the proposals that you do not support (reject).

**Colofon**

The assessment form and the guide for patient reviewers and for organizations are produced under supervision of the committee for participation of the Collaborating Health Foundations (SGF).

Use and adjustment of this form are allowed with acknowledgement of the source. For information or questions, e-mail to: secretariaat@gezondheidsfonden.nl
