# Supplementary material for: Development of a standard form for assessing research grant applications from the perspective of patients
Source: Res Involv Engagem. 2018 Sep 3;4:27. doi: 10.1186/s40900-018-0112-4 (PMC6120065; doi:10.1186/s40900-018-0112-4)
Supplement: Supplementary file 3 — English guide for organisations. (PDF 310 kb) [file 40900_2018_112_MOESM3_ESM.pdf]

Guide for organisations

Introduction to the use of the SGF form for the assessment of research grant applications from the patients' perspective

This guide describes the use of the SGF form for the assessment of research proposals by patient reviewers.
It is based on the feedback from a broad group of stakeholders obtained during several workshops and workshops and training courses, and meetings of the SGF committee for participation and a survey among patient reviewers of SGF member organisations and the Patient Federation Netherlands.

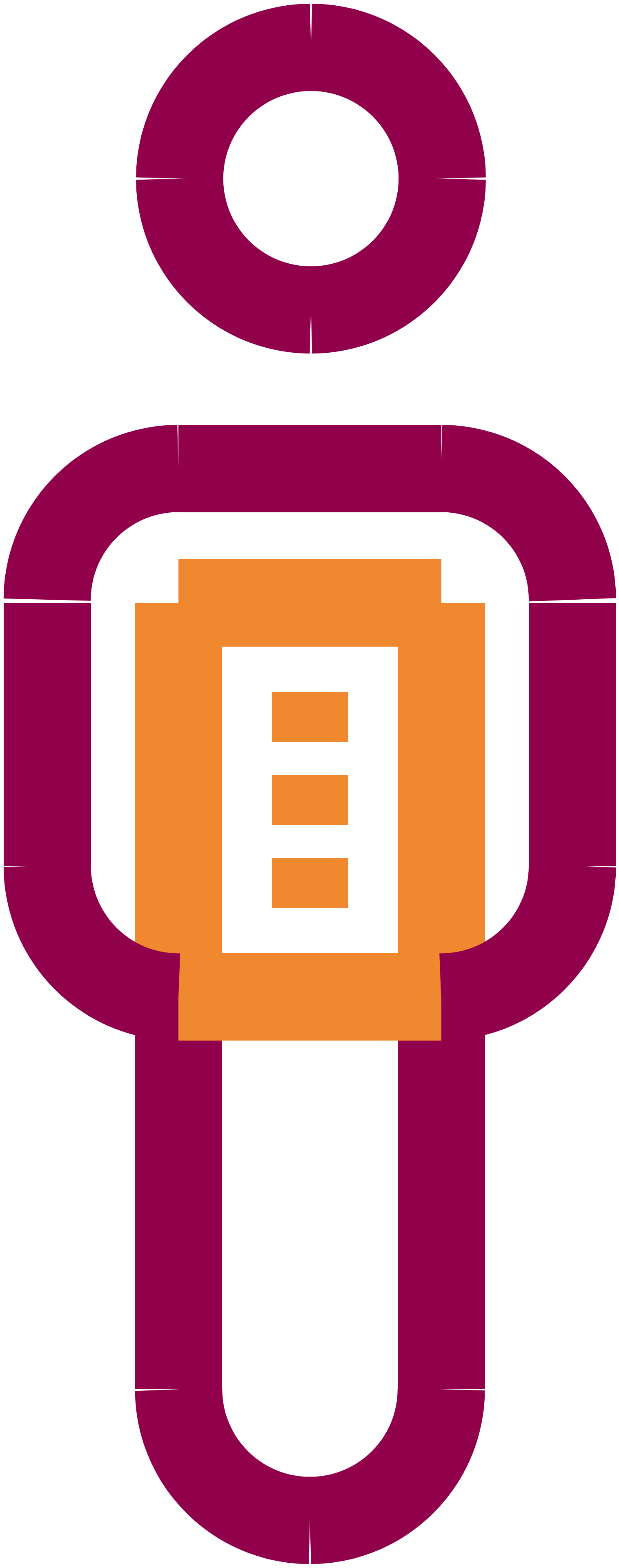

Samenwerkende GezondheidsFondsen
SGF

This guide contains tips and points to consider for the use of the SGF form and is primarily intended for people who are responsible for the preparation and implementation of assessment forms. The purpose of the form is to provide research sponsors, research institutions and patient advocacy organizations in the Netherlands a tool to support the assessment of research proposals from a patients' perspective. The form is drawn up in such a way that it enables customization within organizations or research programs. This questionnaire was developed to have research requests assessed in a more uniform way by patient reviewers. This form is currently being implemented by several organizations and will be evaluated after one year. Any feedback from your experience as a user and additions to the content can be sent to secretariaat@gezondheidsfondsen.nl

Producing the form

The SGF form 'assessing research applications from a patients' perspective' is a generic list of options for questions based on existing assessment forms. The form builds on model developed by Truus Teunissen et al. and other scientific sources. Every health research fund will have to make its own choice which questions and which associated terminology are relevant for a specific research call. Every fund will also have to decide for themselves which persons are eligible to represent the patients' perspective.

In this guide a suggestion is given which questions are recommended (⍰) and which are optional (⍰), that is, depending on the type of research (see appendix in this guide). Here we distinguish between fundamental research, translational research, clinical and applied research, social science and implementation research and a project idea.

Each fund will also have to consider the weighting of the different assessment categories. So may the risks (category 4) and the burden on participants (category 5) weigh more heavily in clinical studies than in fundamental research. We have not made a distinction between different diagnoses or conditions. It is up to each fund, preferably in dialogue with its own patient or advocacy organization(s), to arrive at a final form. In this guide we give examples of the choices that can make regarding certain questions, terms and rating scales.

Finally, we deem it necessary that every patient reviewer is explicitly asked about personal involvement with the research proposal to ensure a fair and transparent assessment process from the perspective of patients. After all it is a shared responsibility to avoid any appearance of a potential conflicts of interest. This also includes the call to patient reviewers to respect the confidential nature of the research application. Both aspects can also be part of a training or instruction session.

Selecting questions

Lay sumary

When developing the SGF form for research grant assessment, and when writing the brochure for patient reviewers, we assumed that researchers write a comprehensive public summary in Dutch. This summary is primarily intended for patient reviewers and is preferably written in a language that is readable for a broad group of people, i.e. without jargon. This summary preferably follows a standard format.

Some health funds use a standard format for the public summary that must enable patient reviewers to come to a judgment from a patient' perspective. Which questions are relevant can vary per condition or research call. We provide a few examples:

Basic research

In fundamental research, the demand for outcome measures (2.8), burden on participants (category 5) or inclusion and exclusion criteria (8.2) may be less important (or even not applicable) than in a program with a lot of clinical research.

PIF

If the METC has already approved an investigation, it is less relevant to have the PIF assessed by patient reviewers. After all, the researcher is no longer allowed to change the PIF.

Formulating questions

Every health research fund can adapt the formulation of questions to their own target group or research programme.

For example, it may be desirable to formulate the question about 'extension of the expected lifespan' (2.5) differently for research into life-threatening diseases. Then, for example, it is more common to speak of "improving the chance of survival". Within mental health care it may be relevant to ask "does the research lead to less suicides".

The question "improves the self-efficacy (autonomy) of patients" (2.2) can also be adapted to the target group within mental health where the concept of recovery is more prevalent.

Scoring

Research among panels of patient reviewers has revealed that they clearly prefer a 4 or 5 point scoring scale. Less is seen as undesirable, more as meaningless. Furthermore, it is recommended that peer reviewers (other researchers) use the same scale or ranking for their final judgement (fellow scientists) as patient reviewers. Assessment committees and boards can then better compare the judgments of both parties.

Weighting of categories

The survey among panels of patient reviewers has also shown that all those involved are almost unanimous in favor of weighing categories when calculating a final judgement. This weighting differs from fund to fund and will have to be determined per program call or organization. The weighting is preferably done in consultation with the panel of patient reviewers, for example with the aid of a simple prioritization questionnaire.

Terminology

The form assessing research applications from patients' perspective uses the term patient, patient organization, patient representative and patients' perspective. If you use questions from this form, we advise you to replace the term patient with a terminology appropriate to your objective or research program. This can be done by replacing 'patients' with 'children with diabetes', 'people with depression' or 'elderly / people over 60 years'. For example, in the case of research in the area of prevention, 'patients' can be replaced by 'people from the target group' or 'the public'.

To give more clarity about the use of the different terms to indicate the target group, we have drawn up a number of definitions. Figure 1 shows which roles can be distinguished from the patients' perspective when assessing research applications and where overlap is possible. This figure also gives you guidance in determining the persons that are eligible within your organisation to represent the patients' perspective when assessing research grant applications.

.

Nomenclature for direct and indirect patient involvement

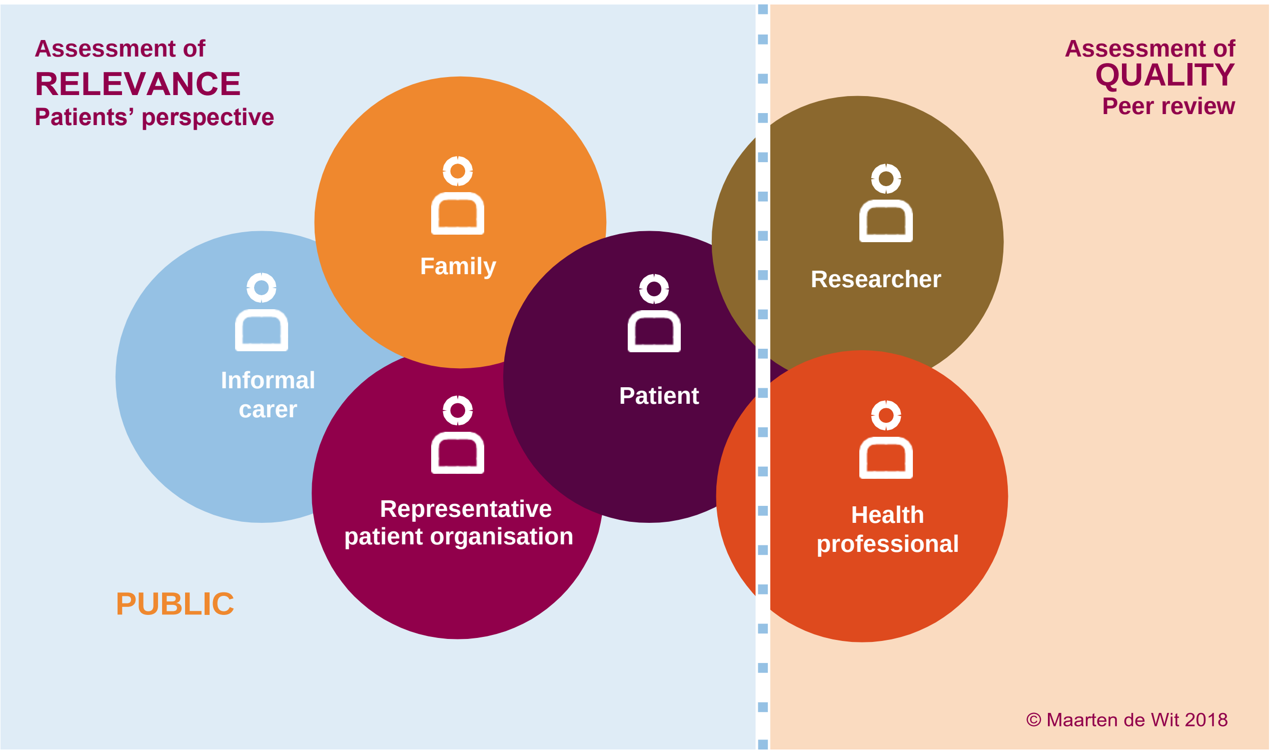

Figure 1

Patients' perspective

The patients' perspective can be integrated in scientific research in two manners, preferably applied simultaneously:

1. **Consultation** - Individual participation of patients or their representatives without having influence on the goal and design of the research. The involvement takes place in the context of collecting data. That is why there is one-way communication: from the patient to the researcher. This form of participation can take place in the role of, for example, a participant in a study (often referred to as 'subject') or respondent.

2. **Collaboration** - Collective representation of interests by patient experts or patient representatives who are involved in formulating the goal and design of the study. There is a partnership in which communication runs in two directions. This form of participation can take place in the role of, for example, research partner, patient reviewer or advisor.

Patient reviewer:

The person who assesses scientific research applications from the perspective of the target population. That can be patient, but also a family member, an informal carer or a patient representative.

Lay person:

In the form and in the guides we avoid the term ‘lay’. Some research programmes speak about involvement of the ‘public’, for example in the case of research in the area of prevention or diagnostics. This term is a synonym for lay, but more common in Dutch language.

Informal carer:

Important person from the social netwerk of the patient who regularly provide care, whether or not paid and whether or not with an appropriate education in health care.

Family:

Important person in te direct environment of the patient, most often a partner or close relative.

Patient:

A person with personal, first hand experience of a particular disease or condition as well as its impact on daily life and health care. Depending on the context, different terms can be used as synonym such as clients, elderly people, service users, end user or consumers.

Patient involvement:

Representing the patients’ perspective into scientific research on behalf of the target population. When patient experts participate in scientific research, we speak of direct patient participation. When family members, informal carers or representatives of an advocacy group (patient representatives) participate in scientific research on behalf of the target population, we speak of indirect patient participation.

Patient representative:

A person who is committed to the collective advocacy for a certain target group. Figure 1 shows which people can take on this role: patient experts, family or close relatives, informal caregivers or representatives of a special interest organization.

Public:

A representative of the public is someone who participate in research with the purpose to ensure societal, disease or condition transcending interests. This can be done on behalf of an advocacy organisation, but also from a personal commitment or interest to contribute to society. Representatives from the public can be asked in the case of research around topics such as ethical issues, prevention or screening programmes.

Training and support

Patient reviewers that use this form will benefit from good support. The SGF guide for patient reviewers is part of this. But they also benefit from a good oral introduction or training before the start of their task as evaluators of research proposals. Such an introduction not only increases the motivation and involvement of the patient reviewers, but also contributes to a more uniform application of the assessment form and procedure. For example, attention can be paid to themes such as 'communication', funding streams in health care research, structure of a research application and the role of a Medical Ethics Committee. In the Netherlands training for patient reviewers is, among other things, given by PGOsupport.

SGF form for the assessment of grant applications by patient reviewers

(see separate file “SGF Form for organisations English”)

Colofon

The assessment form and the guide for patient reviewers and for organizations are produced under supervision of the committee for participation of the Collaborating Health Foundations (SGF).

Use and adjustment of this form are allowed with acknowledgement of the source. For information or questions, e-mail to: secretariaat@gezondheidsfonden.nl
