# Supplementary material for: Development of a standard form for assessing research grant applications from the perspective of patients
Source: Res Involv Engagem. 2018 Sep 3;4:27. doi: 10.1186/s40900-018-0112-4 (PMC6120065; doi:10.1186/s40900-018-0112-4)
Supplement: Supplementary file 4 — English standard form for patient reviewers. (PDF 227 kb) [file 40900_2018_112_MOESM4_ESM.pdf]

Standard form for the assessment of research grant applications from the patients' perspective

This form comes with a brochure in which the questions are explained. We advise you to familiarize yourself with this information before filling in the assessment form. Italic words are described in the brochure.

Good (G) You are satisfied with information in the application;
Sufficient (S) Some information is lacking or you wish some minor adjustments or clarification;
Moderate (M) Much information is lacking or you wish some major adjustments or clarification;
Insufficient (I) You are not satisfied with the information in the application because important information is lacking or because you think the information is not correct.

Are you in any way involved in this grant application? Yes / No

If you have answered 'yes' you should consider whether you are sufficiently able to objectively assess this research application. We also ask you to always respect the confidentiality of the document.

**1. Lay summary**	G	S	M	I	n.a.
1.1 Is the summary of the research application written clearly?					
1.2 Explain your assessment					
**2. RELEVANCE for the TARGET GROUP**	G	S	M	I	n.a.
2.1 Does the research address the needs of patients and/or their *carers* (now or in the future)?					
2.2 Does it improve self-efficacy (autonomy) of patients?					
2.3 Does it improve the *quality of life* of patients?					
2.4 Are the results applicable in practice for patients?					
2.5 Does it improve life expectancy?					
2.6 Does it improve *quality of care* for patients and/or carers (now or in the future)?					
2.7 Are the for patients most relevant *outcome measures* included?					

2.8 Do you have suggestions for outcomes or measurement instruments?					
2.9 Explain your assessment					
**3. RELEVANCE for SOCIETY**	G	S	M	I	n.a.
3.1 Does the research improve the *social participation* of patients?					
3.2 Does it contribute to better prevention?					
3.3 Does it contribute to better diagnostics?					
3.4 Does it contribute to more *understanding* for the life with an illness or health limitation?					
3.5 Does it contribute to the control of health care expenses?					
3.6 Explain your assessment					
**4. RISKS for *STUDY PARTICIPANTS***	G	S	M	I	n.a.
4.1 Are the risks for *participants* clearly described?					
4.2 Do you find the risks for *participants* are acceptable?					
4.3 Do you have suggestions to reduce the risks for participants?					
4.4 Explain your assessment					
**5. BURDEN for *STUDY PARTICIPANTS***	G	S	M	I	n.a.
5.1 Is the burden for *participants* clearly described?					
5.2 Do you find the burden for participants is acceptable?					
5.3 Do you have suggestions to reduce the burden for participants?					
5.4 Explain your assessment					
**6. FEASIBILITY of the RESEARCH**	G	S	M	I	n.a.
6.1 Do you think this research can be executed?					
6.2 Is there sufficient *collaboration* with relevant disciplines or stakeholders?					
6.3 Do you expect sufficient people in the target group are willing to take part?					

6.4 Explain your assessment					
**7. PATIENT PARTICIPATION**	G	S	M	I	n.a.
7.1 Have *patient* (*representatives)* been sufficiently involved in the research design?					
7.2 Are patient (representatives) involved during the execution of the research?					
7.3 Do patient (representatives) receive support when participating?					
7.4 Is patient participation included in the budget?					
7.5 Explain your assessment					
**8. REPRESENTATIVITY**	G	S	M	I	n.a.
8.1 Does the research take *diversity* into account?					
8.2 Are the *inclusion and exclusion criteria* clearly explained and justified?					
8.3 Do the involved *patient* (*representatives)* sufficiently represent the target group in this research?					
8.4 Explain your assessment					
**9. ETHICS AND SAFETY**	G	S	M	I	n.a.
9.1 Is the Patient Information Form (PIF) understandable for people in the target group in this research?					
9.2 Is all relevant information for the target group provided?					
9.3 Do study participants have *freedom of choice*?					
9.4 Are privacy, *safety* and access to services for study participants guaranteed?					
9.5 Is there anything missing in the PIF					
9.6 Explain your assessment					
**10. COMMUNICATION**	G	S	M	I	n.a.
10.1 Are study participants sufficiently informed about the progress and results?					

10.2 Will the findings be shared with (future) patients outside the study and/or with the public?					
10.3 Explain your assessment					
**11. IMPLEMENTATION of RESEARCH FINDINGS**	G	S	M	I	n.a.
11.1 If positive, are the results expected to be well implemented in practice?					
11.2 Does the proposal comprise a plan or approach for implementation or follow-up to make the results usable in practice?					
11.3 Explain your assessment					
**12. FINAL JUDGEMENT**					
12.1 Wat is your advice regarding this research proposal?<br>☐ Approve<br>☐ Approve with conditions<br>☐ Reject<br>☐ No assessment possible					
12.2 Explain your final judgement / advice					
12.3 What recommendations or suggestions for improvement would you like to give to the researchers to optimize the research from the perspective of patients?					
